# Miniaturization of Non-Assembly Metallic Pin-Joints by LPBF-Based Additive Manufacturing as Perfect Pivots for Pantographic Metamaterials

**DOI:** 10.3390/ma16051797

**Published:** 2023-02-22

**Authors:** Florian Gutmann, Maximilian Stilz, Sankalp Patil, Frank Fischer, Klaus Hoschke, Georg Ganzenmüller, Stefan Hiermaier

**Affiliations:** 1 Department of Sustainable Systems Engineering—INATECH, Albert-Ludwigs-University Freiburg, Emmy-Noether-Straße 2, 79110 Freiburg, Germany; 2 Fraunhofer Institute for High-Speed Dynamics (EMI), Ernst-Zermelo-Str. 4, 79104 Freiburg, Germany

**Keywords:** additive manufacturing, joint design, non-assembly, metamaterial, pantographic structure, metals, LBPF, miniaturization

## Abstract

This work introduced additively manufactured non-assembly, miniaturized pin-joints for pantographic metamaterials as perfect pivots. The titanium alloy Ti6Al4V was utilized with laser powder bed fusion technology. The pin-joints were produced using optimized process parameters required for manufacturing miniaturized joints, and they were printed at a particular angle to the build platform. Additionally, this process optimization will eliminate the requirement to geometrically compensate the computer-aided design model, allowing for even further miniaturization. In this work, pin-joint lattice structures known as pantographic metamaterials were taken into consideration. The mechanical behavior of the metamaterial was characterized by bias extension tests and cyclic fatigue experiments, showing superior levels of performance (no sign of fatigue for 100 cycles of an elongation of approximately 20%) in comparison to classic pantographic metamaterials made with rigid pivots. The individual pin-joints, with a pin diameter of 350 to 670 µm, were analyzed using computed tomography scans, indicating that the mechanism of the rotational joint functions well even though the clearance of 115 to 132 µm between the moving parts is comparable to the nominal spatial resolution of the printing process. Our findings emphasize new possibilities to develop novel mechanical metamaterials with actual moving joints on a small scale. The results will also support stiffness-optimized metamaterials with variable-resistance torque for non-assembly pin-joints in the future.

## 1. Introduction

Mechanical metamaterials are lattice structures with a macroscopic mechanical behavior that is governed by a specific unit cell geometry, rather than the intrinsic material properties of the base material used to fabricate the structure [1,2,3,4,5,6]. For instance, mechanical metamaterials can twist under pressure loading [7], exhibit a negative Poisson’s ratio [8], present exceptional elastic deformation [9], and have a negative stiffness [10]. The kinematic degrees of freedom of the unit cell are thus of paramount importance for the behavior of the metamaterial. This work introduced a concept for realizing true rotational joints into the unit cell, facilitating metamaterials with qualitatively new features.

The complex structure makes it challenging to manufacture functional metallic metamaterials whereby additive manufacturing (AM) can offer a solution for the fabrication of complex shapes. In particular, laser powder bed fusion (LPBF) is an AM process that allows the manufacturing of complex metallic structures. However, it is common. and often necessary, to add support structures to surfaces with an angle of 45° or less to the build plate. Comparisons between printed geometry and its nominal computer-aided design (CAD) models show structural distortions and geometrical errors [11,12,13]. The accuracy of the additive manufactured parts is influenced by thermal stress, process parameters and surface roughness. For filigree structures, the surface roughness is primarily defined by partially fused particles that stick to the molten metal [14]. These deviations are critical for geometric functionality and the assemblability of additive manufactured parts [15]. Therefore, to directly print non-assembly metamaterials successfully, a high accuracy of the print compared to its CAD is required.

In this article, the metamaterial pantograph (a pantographic sheet structure) was directly manufactured as a non-assembly (not requiring an additional assembly step) structure with high-quality, true rotational pin-joints (perfect pivots) using LPBF. A metallic pantograph with rigid-joints as pivots can reach large deformations and is reliable against constructive defects such as structural imperfections and therefore constructive defects do not alter the overall behavior of the pantograph [16]. Furthermore, in this metamaterial, the deformation energy also depends on the second gradient of the displacement. This dependency is maximized if the pivots are perfect, meaning real pin-joints in a non-assembly that allow free rotation. Thus, there is no deformation energy in the pivots but the bending energy (second gradient) in the beams can be observed [17,18]. By optimizing the stiffness distribution of each pivot, variation of the rigid joint, quasi-perfect to perfect pivots of the pantograph, the maximal elongation of this structure can be increased [19].

Several studies focus on the design of joints optimized for the LPBF printing process. In particular, the combination of concave and convex shapes for the rotator and stator of a pin-joint appear very promising since the structures are self-supporting during the printing process and do not need support structures inside the joint clearance [20,21,22]. Furthermore, it was shown that large joint clearances should be avoided since they lead to vibrations and instabilities of realized joints, non-assemblies, and their functional structures [23,24]. Boschetto et Bottini [22] and Su et al. [23] presented pin-joints that were printed at an angle to the build plate. They pointed out that a reduction of the maximal particle size of the manufacturing powder and an optimization of the process parameters are necessary to reduce the joint clearance and obtain a higher quality joint. Notably, the angled joints show a warpage in build direction, and can result in a merging of the separate parts of the joint. In contrast to expectations, angled printing reduced the number of areas that are critical during manufacturing and resulted in smaller realizable joint clearances compared to printing joints in 90° to the build plate.

The main focus of the present study was to successfully manufacture miniaturized pin-joints using especially developed and optimized process parameters for titanium alloy (Ti6Al4V) on a standard LPBF machine. The process optimization removed the need to compensate the CAD model geometrically, thus enabling the miniaturization of the pin-joints. Thereby, the aim was to realize high-quality pin-joints (small joint clearance, torque free rotation, no instability of the joint) while printing the joints in a 45° angle to the build plate. To achieve this goal, a concave-shaped pin and a convex-shaped hole for the pin-joint were straightened out to reduce the number of different angled surfaces during the printing process. The joint clearance was set to approximately twice the maximal particle size of the used powder to avoid instability of the joint. Furthermore, the miniaturized pin-joints were used as perfect pivots for pantographic metamaterials. The miniaturization was compared to previous manufacturing of pantographic metamaterials with pin-joints [25] using standard process parameters provided by the manufacturer.

## 2. Materials and Methods

### 2.1. Pin-Joint and Pantographic Metamaterial Design

**Figure 1:**
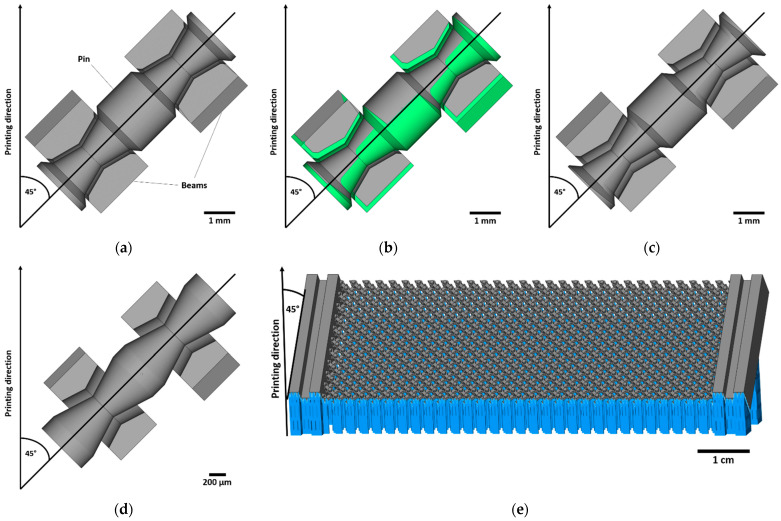
Pin-joint design and adaption for the miniaturization. The pin-joint design is a combination of concave-shaped (also known as drum-shaped) edged holes and convex-shaped edged pins: (**a**) uncorrupted and non-miniaturized CAD model in a 45° angle to the build plate. The smallest angle to the build plate within the joint is 30° and the clearance in the pin-joint is 120 µm; (**b**) marked surface areas for geometrical compensation of 200 µm in building direction; (**c**) CAD model after geometrical compensation to be used for printing; (**d**) simplified version of the previous pin-joint with downscaling of its dimensions and without geometrical compensation. The smallest angle of down facing surfaces towards the build plate within the joint thereby is 27.5°. The height of the joint was reduced to 2.6 mm; the dimensions of the resulting metamaterial (**e**) with the clamping structure are 87.1 × 26 × 6 mm^3^.

The pin-joint design is an adaption to the self-supporting pin-joint [20] with concave-shaped hole and convex-shaped pin. Compared to the published design, the curved shapes are edged to simplify the model and reduce the number of different angles within each joint for downscaling purposes. Figure 1 shows the two pin-joint designs (a,d) used to manufacture variants of pantographic metamaterials. A limiting factor for the downscaling of the joint is the necessary diameter difference of the pin compared to the applied clearance combined with the minimal hole diameter. In this context, the minimal angle of any surface of the pin-joint to the build plate defines the necessary height of the joint to guarantee the diameter difference.

The first pin-joint model (a) is designed to be manufactured using established process parameters for bulk materials. These process parameters exhibit a laser melt track depth of around 200 µm. Therefore, surfaces facing downwards to the build plate are geometrical compensated by 200 µm regarding build direction (commonly known as z-compensation) to retain the joint’s circularity when printed in a 45° angle. The modeled joint clearance was set to 120 µm to prevent potential merging. In Figure 1a–c the process of geometrical compensation is demonstrated, whereby (a) is the initial CAD model, (b) shows the to be removed marked structure and (c) the final CAD model used for the printing. The dimensions of the pantographic metamaterial with these pin-joints (total of 117) are 90.2 × 30 × 8 mm^3^ wherein the beams had a square section of 2.2 × 2.2 mm^2^. The total height of the pin-joints was 7 mm with a diameter of 0.8 to 2 mm.

Contrary to this, for the second pin-joint variant, (d) in Figure 1, optimized process parameters are used to obviate the need for geometrical compensation. By removing the need to compensate the CAD model by 200 µm, smaller pin-joint models can be realized without corrupting it due to geometrical changes thus enabling their miniaturization. The joint clearance is again set to 120 µm. The square section of the beams is reduced to 0.6 × 0.6 mm^2^ and an enlargement around the joints is added keeping the square sections constant. The total height of the pin-joints is reduced to 2.6 mm and their diameter to 0.35–0.67 mm. The dimensions of the pantographic metamaterial are kept relatively close to the previous one (87.1 × 26 × 6 mm^3^) including a total of 619 single pin-joints. Furthermore, a pantographic metamaterial with the same dimensions but with a clearance of 0 µm (rigid-joints) are manufactured for comparison.

### 2.2. Additive Manufacturing

The samples are produced on an EOS M 100 LPBF machine (EOS GmbH, Krailling, Germany) with a 200 W laser unit (YLR-series, CW-laser, wavelength 1070 nm) that has a focus diameter of 70 µm. The layer thickness is set to 20 µm. The titanium alloy powder used for the manufacturing consists of used powder sieved with a 63 µm mesh, where the consumed powder of the previous print is replenished with fresh powder. The authors expect no significant effect by using the Ti64 powder in sieved condition which is supported by the study of Sukhov et al. [26] about the 16-fold recycling of a nickel alloy powder. To avoid oxidation, an argon-based inert gas atmosphere of O_2_ < 0.1% is applied. All pin-joints and the pantographic metamaterials orthogonally aligned to the joints are printed in a 45° angle to the build plate. Each body of this non-assembly has a separate support structure and is illustrated in Figure 1e.

The first non-miniaturized joints and pantographic specimens are manufactured using manufacturer process parameters for producing dense bulk material. For the miniaturized pin-joints an optimized process parameter set is developed, based on the methodology developed in Pfaff et al. [27,28], and used for manufacturing. An iterative design of experiments (DoE) with single laser track experiments is carried out to find process parameters that allow the realization of a small melt track size. As Khorasani et al. [29] point out the codependency of the process parameters and thermal dependent laser absorptivity of the powder on the resulting melt track dimensions, the selection of the process parameter requires finetuning. Thereby, the goal is to minimize the size of a single melt track, respectively melt width and melt depth. The size of a single melt track is determined by measuring the dimensions of a specimen printed only with single laser tracks containing an overhanging structure element. Based on these process parameters the hatch distance is also iterative adapted to achieve a high density. The optimized process parameter set is summarized in Table 1.

By reducing the additive building blocks (melt tracks) on standard LBPF machines, the geometrical accuracy of the additive manufactured miniaturized pin-joints should be improved. The parameter set is split in skin and core parameters. The core parameters are applied if a laser exposure track is positioned geometrically so that the resulting bigger melt track stays within the outer shape of the underlying CAD model. Therefore, the number of necessary laser exposure tracks is reduced, and productivity is increased while keeping the previous mentioned geometrical accuracy.

The outer surfaces of all specimens were sandblasted to remove adherent powder particles. Further surface treatments were not conducted. All specimens were examined without any heat treatment.

### 2.3. Powder Analysis

The Ti64 powder (EOS Titanium Ti6Al4V, EOS GmbH, Krailling, Germany) used for manufacturing was analyzed on a CAMSIZER^®^ X2 (Micotrac MRB, Haan, Germany). The setup offers a measurement range of 0.8 µm to 8 mm. The dynamic image analysis (ISO 13322-2) was conducted with a dispersion pressure of 30 kPa. Thus, a volume distribution was computed.

### 2.4. CT Scan

A high-resolution X-ray micro-computed tomography (µ-CT) of a miniaturized pin-joint is conducted on a SkyScan 1272 (Bruker, Germany) at 100 kVp with a XIMEA xiRAY16 detector. Measurements are done using grey-scale analysis and circle-fit function of Image J (version 2.3.0/1.53q) in correlation with an effective image pixel of 1.5 µm.

### 2.5. Mechanical Testing

For mechanical testing, the pantographic metamaterials (0 µm and 120 µm joint clearances) manufactured with the optimized process parameters were pulled in its bias direction using a Zwick Z100 testing machine (ZwickRoell AG, Ulm, Germany) in displacement control mode. This bias-extension test was conducted with a velocity of 9.364 mm/min, a strain rate of approximately 0.002/s until an elongation of 25% was reached. Furthermore, cyclic fatigue testing was performed on the same testing setup for the 120 µm joint clearance pantographic metamaterial. The velocity for the cyclic fatigue testing was increased to 93.64 mm/min for a distance of 0 to 15 mm which approximately equals 20 % maximal elongation per cycle. The specimen was cycled for 100 cycles.

### 2.6. Simulation

Simulations of the bias-extension tests were carried out using the second gradient continuum model from Giorgio et al. [30] within the author’s finite element method (FEM) code relying on B-spline interpolation. The following parameters were used: beam width and height of 0.6 mm, pivot diameter of 0.6 mm, pivot height of 1.2 mm, pivot distance of 1.77 mm and a Young’s Modulus of 90.0 GPa.

## 3. Results and Discussion

### 3.1. Powder Analysis

**Figure 2:**
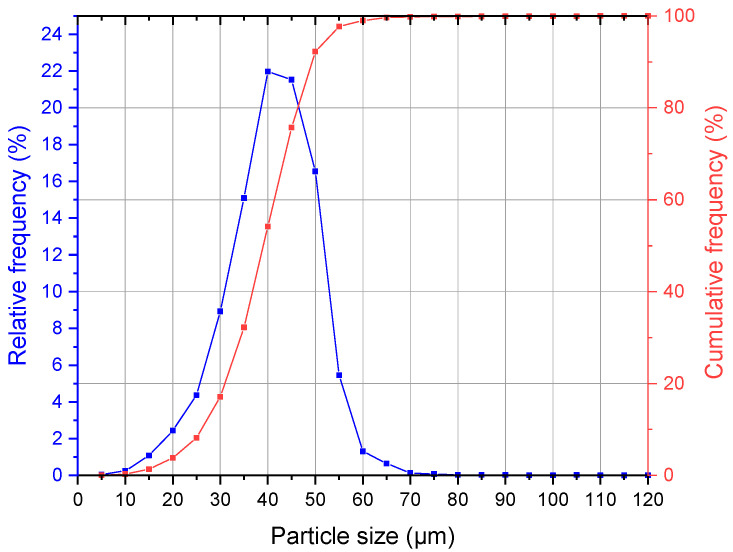
Particle size distribution of the used Ti64 powder in sieved condition.

The particle size analysis of the Ti64 powder shows a cumulative particle size distribution of D10: 26.22 µm, D50: 39.04 µm and D90: 49.21 µm, the full measurement is illustrated in Figure 2. The particle shape analysis calculated a width/length ratio (aspect ratio) b/l3 of 0.8523, a specific surface area S_v_ of 165.541/mm and from the area-to-perimeter ratio a mean sphericity SPHT3 of 0.8672, for a perfect sphere this value is equal to 1.

### 3.2. Additive Manufacturing and CT

**Figure 3:**
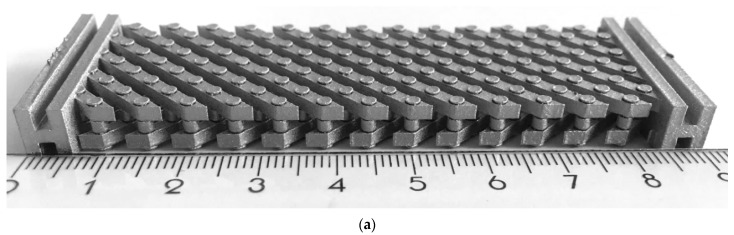

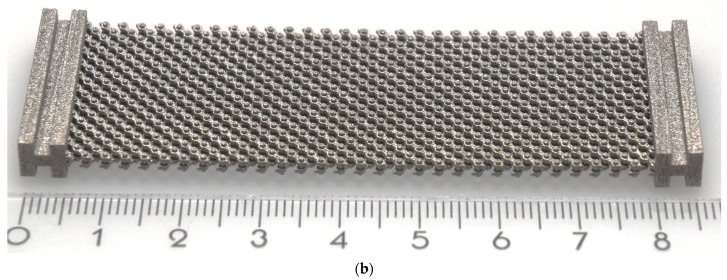
Comparison of printed pantographic metamaterials with pin-joints, perfect pivots next to a metric ruler in centimeter: (**a**) pantographic metamaterial with non-miniaturized pin-joints; and (**b**) the pin-joints were miniaturized and their number in the metamaterial is increased by a factor of five.

The main focus of the present study was to demonstrate the successful manufacture of miniaturized pin-joints in metamaterials with the particular challenge of printing the pin-joints in a 45° angle to the build plate. Therefore, the metamaterials shown in Figure 3 were realized. The first pantographic metamaterial (a) exhibits free moving pin-joints with backlash. The joint clearance is measured to be 125 µm. This pantographic metamaterial will serve as the basis for the miniaturization shown here and will not be mechanically analyzed in this work. Local–global digital volume correlation analyses for in situ torsion of this non-miniaturized pantographic metamaterial can be found in Valmalle et al. [25].

**Figure 4:**
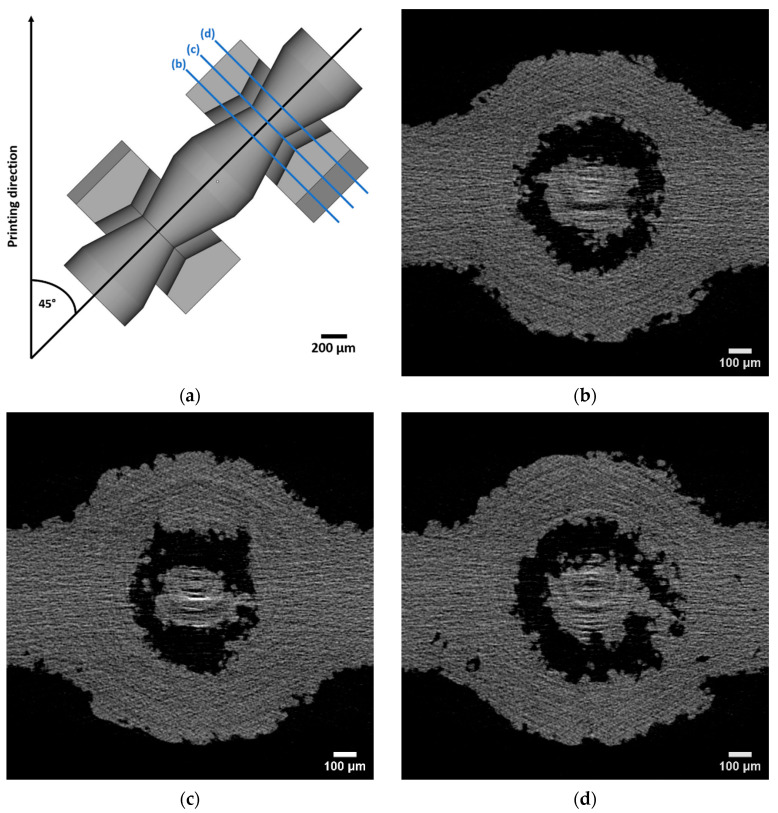
CT images of a pin-joint with a pixel size of 1.5 µm conducted on a SkyScan 1272: (**a**) shows the selected sections (**b**–**d**) for the CT imaging parallel to the beam plane with an averaged distance of 150 µm between the shell of the beam and these sections. The left side of the CT images point towards the building direction. The joint clearance measured by the difference of the radius using circular fit of the pin and the beam hole is (**b**) 115 µm, (**c**) 127 µm and (**d**) 132 µm.

The metamaterial realized with the miniaturized pin-joints (b) exhibits backlash primarily along the pin’s axis. Here, the joint clearance was measured to be on average 126 µm. In the CT scan, as seen in Figure 4, the clearance is not uniform. At surfaces facing towards the build plate, the clearance is locally smaller and shows possible merging or touching of the pin with the beam. These pin-to-beam contacts are not consistent throughout the total height of the pin-joint. Furthermore, the size and morphology of the structures visible in the clearance area of the pin-beam contact look like adhering or partly melted powder particles. Hence, a particle analysis of the powder used for manufacturing is conducted to put this assumption into perspective. Accordingly, two adhering powder particles of the size of the sieve mesh could fill the clearance. A potential error propagation originating from the CAD model or underlying stl file could also explain this pin-beam contact, but since the final slice file presents no flaws in the printing software this error source is being excluded. Another option is that the afore mentioned surfaces with an angle of 27.5° to the build plate present a poorer surface quality compared to the other surfaces of the part. An offset or porosity on these surfaces with a diameter of two to three D50 particle size could also fill the clearance and result in the observed merging or touching. Combining these ideas with the previously witnessed backlash of the whole metamaterial, the touching of pins with beams are more likely than merging.

**Figure 5:**
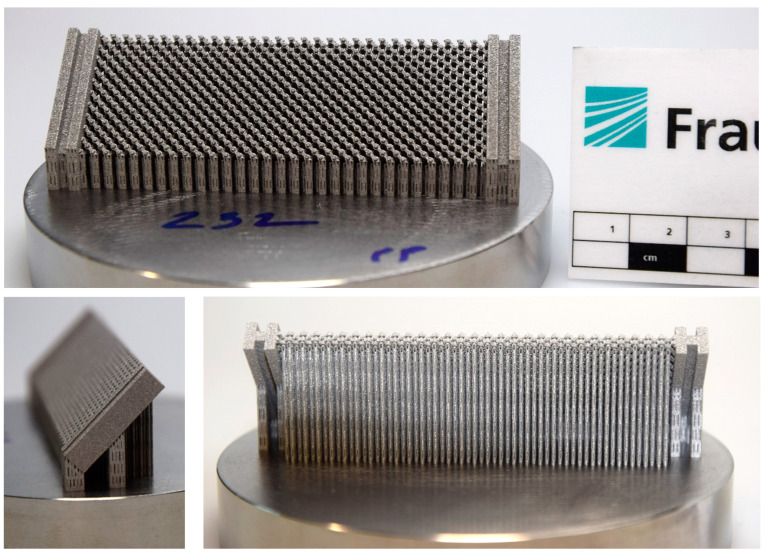
Front, side and back view of the miniaturized as-built manufactured pantographic metamaterial with support structures for every separate body (each pin-joint, most beams and the clamping) on a Ø 100 mm build plate.

In Figure 5 the printed metamaterial is shown in different views with its support structures connecting it to the build plate. When removing the part from the build plate or removing the support structures from the metamaterial it can happen that a part of one or more pins, sometimes even beams get removed together with the support structure. Although the optimized process parameters allow the realization of potential smaller pin-joints, the post processing, especially the removal of support structures from the pin of this multi-body-system, remains a challenge and can result in damaged specimens. An optimization of the process parameters used for printing the support structure or of its geometry, mainly the teeth-structure connecting the support and the actual part, can potentially increase the chance of a damage free support removal. Here, the size of the pin-joints are kept at the previous mentioned dimensions and as illustrated in Figure 1. The support structures are frequently successfully removed from the specimen by first removing the specimen and its support structure from the build plate with a diamond band saw. After that, every single support structure is manually removed with a small side cutting nipper while avoiding the application of powerful forces. For the following mechanical testing, only undamaged specimens were selected.

### 3.3. Mechanical Testing

**Figure 6:**
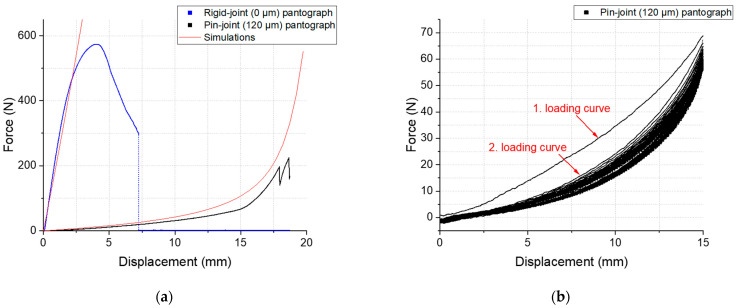
Force-displacement curves of the manufactured pantographic metamaterial specimen with rigid-joints (blue curves), 120 µm clearance pin-joints (black curves) and their associated simulations (red curves): (**a**) shows a bias-extension test conducted for both specimen until an elongation of 25% was reached; and (**b**) shows the cyclic fatigue behavior of the pin-joint pantograph for 100 cycles to an elongation of 20%.

To complement and verify the previous findings, mechanical tests were performed on this pantographic metamaterial with a clearance of 120 µm and on a rigid version of the same metamaterial. The second is identical to the first except the clearance is set to 0 µm to force a merging of beam and pin, a rigid-joint pantograph. The latter has been the subject of many studies on the pantographic metamaterial to date because no perfect pivots were available. The force–displacement curves of the bias-extension and fatigue testing are presented in Figure 6.

In this metamaterial the difference an actual moving joint can make becomes evident. The course of the curve in bias-extension, Figure 6a, changes drastically. It exhibits a completely different mechanical material behavior. The rigid-joint pantograph metamaterial shows an elasto-plastic behavior with strain hardening until a maximum force is reached and structural damage accumulates till failure. In contrast, in the bias-extension test of the pantographic metamaterial with pin-joints a mechanical behavior can be observed, that is primarily affected by the bending energy of the beams. This is of particular importance for studies in the field of higher order continua where a second gradient term for deformation energy is dominant [17,18]. This behavior is explained due to change of the dominant mode of loading. The mode changes from torsion of the rigid-joint’s pivots to a compliant bending mechanism if there is a functional rotational joint as the pivot. The required force for deformation of this pin-joint pantograph stays low compared to the rigid version and reaches 197 N at a displacement of 17.95 mm that equals an elongation of 24% when the first partly failure of the metamaterial is observed. A second partly failure is observed at a force of 224.5 N and a displacement of 18.67 mm (~25% elongation) before the testing stopped.

The simulations showed good agreement to the rigid-joint bias-extension test up to plastic deformation, which was not being factored in. The deviation for the pin-joint bias-extension simulation may be due to the geometries of the beams: at the pivots, the beams are enlarged with a hole in the center where the pin-joint with a 120 µm clearance is located. This change in beam geometry is in the same length scale as the distance between pivots while the continuum model assumes a homogeneous beam cross section. A refined model or a fitting of the cross section to the experiments are expected to bring better results.

**Figure 7:**
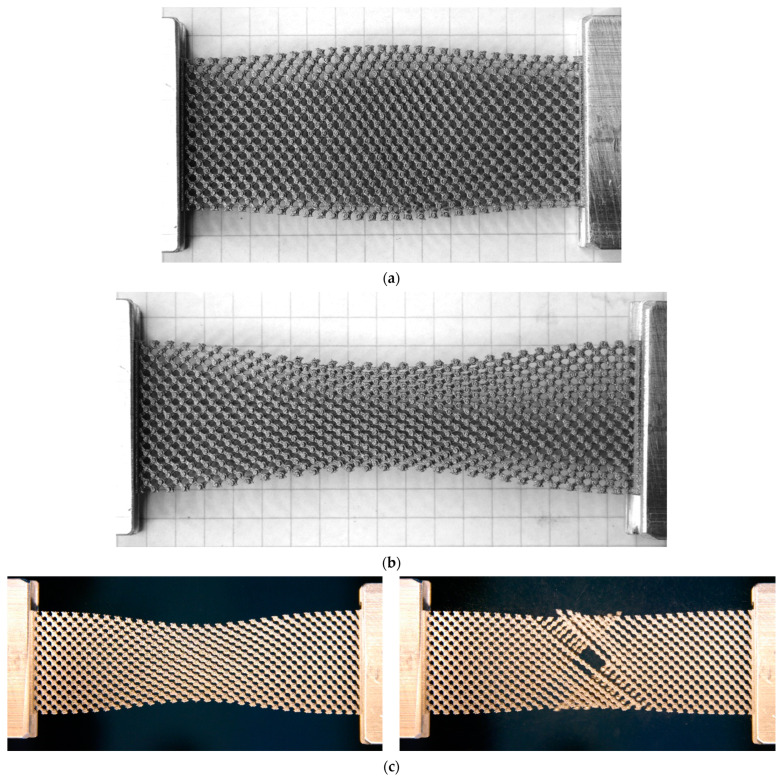
Mechanically deformed pantographic metamaterials with and without pin-joints. Manually pushed by ~7 mm (**a**); and pulled by ~15 mm (**b**) pin-joint pantographs. Images of before and after (**c**) failure of the rigid-joint pantograph bias-extension test.

In Figure 6b, the cyclic fatigue behavior of the pin-joint pantograph is illustrated in a force-displacement diagram. Here, the first loading curve shows a significant higher slope compared to the following 99 cycles, that show rather stable loading and unloading curves with a small hysteresis. The total maximal force of 66.03 N is measured in the first loading curve and in the 100th cycle the maximal force for the loading drops to 55.83 N. Figure 7 provides visual demonstration of the mechanical performance of both metamaterial variants tested. It should be noted that the rigid-joint pantograph fails due to torsion of the pivot (the rigid-joint). In the pin-joint pantograph, this failure mode is prevented due to the kinematic added mechanical freedom of the actual rotational joints. An additional cyclic fatigue testing comparison of rigid- to pin-joint pantograph metamaterial for a displacement of 4.83 mm, an elongation of 6.45% can be found in Appendix A.

## 4. Conclusions

This work introduced an approach to successfully print miniaturized pin-joints as perfect pivots. By using optimized process parameters combined with an ideal CAD model, the need for geometrical compensation was circumvented and miniaturization was achieved. The miniaturized pin-joints were integrated in a pantographic metamaterial and manufactured using the LPBF process. The printed metamaterial specimens were mechanically tested to verify the successful implementation.

With the results presented in this work, the authors conclude that the miniaturization of pin-joints and their integration in the pantographic sheet metamaterial printed in a 45° angle to the build plate was successful. The metamaterial’s behavior was primarily affected by the bending energy of the beams. Therefore, the pin-joints presented in this work can be considered as perfect pivots [18] that allow the realization of second gradient metamaterials.

The cyclic performance of the pin-joint pantograph was far superior to the rigid-joint one because the pivots, the pin-joints, rotate instead of torqueing. This advantage makes the metamaterial more resilient, and storing the deformation energy in primarily bending energy increases its durability. Therefore, until the first failure, the pin-joint pantographic structure can be deemed to behave like a compliant mechanism.

The observed warpage in the build direction of the angled printing remains even with the proposed optimization of this work but is not critical for the manufacturing of a constructed joint clearance with twice the maximal powder particle size. A reduction of the warpage and as well as the joint clearance require further investigations.

The next step will be to investigate how much the joint clearance can be reduced using the presented approach for printing miniaturized pin-joints. The focus will be on the performance of a single joints specimen with a varying joint clearance that will be tested on a torsion testing setup [31]. The potential findings of this proposed next step might enable the realization of stiffness-optimized nonlinear pantographic structures [19].

Future work will investigate the reduced but still existing warpage of the pin-joint geometry. A geometrical compensation in the size of the applied layer thickness up to maximal particle size, if it does not corrupt the final CAD model strongly, might be an option to be considered and further investigated. Furthermore, new LPBF process optimization possibilities will be explored, which, with advances in technology, can be considered to smoothen surfaces in the joint and challenge the remaining warpage.

## Figures and Tables

**Table 1:** Optimized printing process parameter set used for manufacturing of miniaturized pin-joints. The measured melt track width and depth of the corresponding parameter sets are also listed.

Parameter Set	Laser Track	Hatch Distance (µm)	Laser Power (W)	Scanning Speed (mm/s)	Melt Track Depth (µm)	Melt Track Width (µm)
Core *	Stripes	40	70	2000	100	110
Skin	Contour (2×)	8	50	2000	30	90
	Stripes	30	50	2000		

* no contour parameter for the core parameter set.

**Figure A1:**
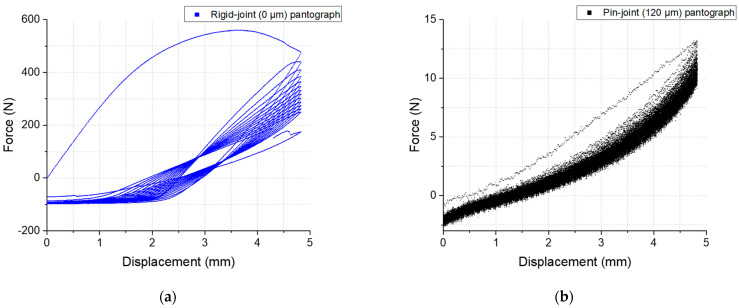
Force-displacement curves of the manufactured pantographic metamaterial specimen in a cyclic fatigue test with an elongation of 6.45% performed for 100 cycles or until failure: (**a**) shows the curve for the rigid-joints pantograph, the structure fails in the 14th cycle; (**b**) shows the curve for the 120 µm clearance pin-joint pantograph without failure.

## Data Availability

Not applicable.

## Appendix A

The difference in mechanical behavior and the successful integration of miniaturized pin-joints are further illustrated in Figure A1, additional results of the cyclic fatigue testing. The rigid-joint pantograph (a) shows in the first fatigue cycle a loading behavior equivalent to the extension test with a maximum force of 560 N, though during unloading the force drops to zero at a remaining displacement of 2.9 mm, indicating the plastic deformation of the first loading. Furthermore, while moving to the starting position a high negative force is measured and it is observed that the structure is plastically elongated and buckles out of plane. With every further cycle there is an accumulation of plastic deformation and damage observed. The structure fails in the 14th cycle.

In comparison, the pin-joint pantograph specimen (b) shows for the same displacement within 100 cycles, no noteworthy fatigue and a maximum force of 13 N with a very small negative resetting force. However, conspicuously is the first loading compared to the rest of the measurement, as a higher force for the displacement is observed. The authors assume, this is due to adhering and residue powder particles in the pin-joint that break free within the first mechanical load as well as the potential local unmerging of pin-beam contacts or moderation of the rough surface that is witnessed in the area of the clearance. Although, the measured and rather low forces compared to the rigid-joint variant indicate no particularly strong merging of pins and beams.
